# Mind-Mindedness and Stress in Parents of Children with Developmental Disorders

**DOI:** 10.1007/s10803-020-04570-9

**Published:** 2020-06-19

**Authors:** Fionnuala Larkin, Marianna E. Hayiou-Thomas, Zaynah Arshad, Matthew Leonard, Frances J. Williams, Nicoletta Katseniou, Rania N. Malouta, Charlotte R. P. Marshall, Maria Diamantopoulou, Etonia Tang, Sneha Mani, Elizabeth Meins

**Affiliations:** 1 Department of Psychology, University of York, Heslington, York, YO10 5DD England; 2 Present Address: York St John University, Lord Mayor’s Walk, York, YO31 7EX England; 3 The Retreat York, York, England; 4 Present Address: The University of Sheffield, Sheffield, England

**Keywords:** Mind-mindedness, Parenting stress, Developmental disorders, Parental attributions

## Abstract

Relations between mind-mindedness (assessed using the describe-your-child interview) and stress were investigated in parents of children with developmental disorders (ADHD, n = 51, ASD, n = 23, Down’s Syndrome, n = 38, and 22q11.2 Deletion Syndrome, 22q11.2DS, n = 32) and typically-developing children (n = 89). Mind-mindedness did not differ across diagnostic groups, and mind-mindedness predicted parenting stress across groups. Parenting stress was lowest in the typically-developing and Down’s Syndrome groups. Across all groups, mind-minded and positive descriptions predicted lower parenting stress, and negative descriptions predicted higher stress. In the developmental disorder groups, describing the children with reference to their disorder was negatively correlated with mind-mindedness. Results are discussed with regard to interventions for families where children have developmental disorders.

Parenting children with developmental and genetic disorders can be a deeply rewarding experience, greatly enriching parents’ lives. Nonetheless, families with a child with a disorder can often face more stressors than families with typically developing (TD) children, such as behavioral difficulties, health concerns, low adaptive functioning, increased contact with health and mental health services, and educational placement difficulties (Blacher et al. 2005). As such, parenting stress has been found to be higher amongst parents of children with genetic and developmental disorders than in those with TD children (Gerstein et al. 2009; Woodman 2014). Although some level of stress is regarded as normative and adaptive for parents (Crnic et al. 2005), the challenges posed by parenting a child with a disorder can be associated with affective disorders in parents (Ingersoll and Hambrick 2011), lower quality of life for families (Lee et al. 2008), and poorer behavioral functioning in children later in childhood, as demonstrated through longitudinal studies (e.g., Neece et al. 2012; Woodman et al. 2015).

However, other variables may protect against developing stress or against the negative consequences of stress. Parents who adopt positive coping strategies like emotional-approach coping strategies (Pakenham et al. 2005) or problem-focussed coping strategies (Smith et al. 2008) report better well-being. Promoting daily positive affect and focussing on positive experiences (Ekas and Whitman 2011; Kayfitz et al. 2010), or nurturing a sense of spirituality (Estes et al. 2009) are also associated with parental well-being.

Amongst parents of TD children, mind-mindedness (Meins 1997)—the parent’s ability to treat their child as an individual with a mind of their own—is an additional factor that appears to protect families against certain negative outcomes. Mind-mindedness in parents of infants up to 12 months is assessed with an interactional measure in which freeplay interactions between parent and infant are transcribed, mind-related comments identified, and the comments coded for whether they are ‘appropriate’ or ‘non-attuned’ depictions of the infant’s internal states. For parents of older children, mind-mindedness is assessed via a brief interview in which the parent is asked to describe their child, with responses coded for the presence of mental attributes (Meins and Fernyhough 2015).

In families from disadvantaged backgrounds, higher maternal mind-mindedness in the first year of life predicted fewer behavioral difficulties in their children during the preschool years (Meins et al. 2013). Parents’ tendency to describe their 4-year-olds with reference to their mental characteristics was negatively correlated with levels of parenting stress (McMahon and Meins 2012). Viewing children as intentional agents, with beliefs and desires, may enable parents to interpret their behavior and thus limit the stress that even challenging or difficult behavior might elicit. Increasingly, parents’ representations of their child and the parent–child relationship have been implicated as important influences on parenting behavior and child outcomes (Dadds et al. 2003; Deater-Deckard et al. 2005; Harrison and Sofronoff 2002).

The present study investigated mind-mindedness in parents of children with developmental disorders and explored its relation with parenting stress in this population. The study included parents of children with Down’s Syndrome (DS), 22q11.2 deletion syndrome (22q11.2DS), Autism Spectrum Disorder (ASD), and Attention Deficit Hyperactivity Disorder (ADHD). There are reasons to expect that being mind-minded towards a child with genetic and developmental disorders may be more challenging than towards a TD child. ASD and ADHD are syndromes largely defined by behavioral disturbance, with additional communication, social interaction, and often learning deficits (APA 2013). Down’s Syndrome and 22q11.2DS are genetic disorders associated with increased behavioral problems and learning disability (Goodwin et al. 2017; Neece et al. 2012), as well as communication difficulties, meaning children may be less able to express themselves and make requests (e.g., Mundy et al. 1995). Consequently, parents may find it more difficult to interpret their child’s moods and behaviors. In addition, children with intellectual/learning disabilities are more likely to exhibit emotional and behavioral difficulties and challenging behavior (Dekker et al. 2002).

Parents’ understanding of the source of their child’s challenging behavior has implications for how well they cope: parents of children with ADHD who viewed their child’s non-compliant behavior as deliberate, rather than due to the child’s inability to comply, experience higher levels of stress (Goldstein and Goldstein 1992). Harrison and Sofronoff (2002) characterised this dichotomy as ‘internal’ versus ‘external’ causes of behavior. Internal causes were ‘his nature’, ‘laziness’, or ‘to get attention’, whereas external causes were seen as ‘the disorder’, ‘medication’, or ‘he doesn’t understand’. Parents attributing challenging behavior to ‘internal’ causes experienced greater stress. However, there may be more complexity to this dichotomy, as these studies did not consider the emotional tone of the parents’ attributions. Where parents can genuinely take their child’s perspective in a developmentally appropriate way, they may be more likely to produce emotionally positive readings of their child’s behavior. It may be that parents’ representations of their child that are both mentalistic and positive confer the greatest protective value (Demers et al. 2010; McMahon and Meins 2012).

Having a positive view of the child’s mental states in developmental disorders may allow parents to frame their actions as understandable or desirable; for example, a child who sleeps poorly might be seen as ‘alert’ or ‘bright’, thus moderating parental frustration (Demers et al. 2010). In contrast, negative representations of the child may give rise to parental hostility—rather than being viewed as alert, the child may be seen as attention-seeking, or purposefully attempting to irritate the parent. It may also be the case that positive, mentalistic representations of the child’s character (e.g., as a fun, sociable, or loving child) buffer parents against the stress imposed by challenging behaviors and other difficulties. In a recent study, Fishburn et al. (2017) found that adoptive parents’ tendency to describe their adopted child largely with reference to placement history was negatively correlated with mind-mindedness, suggesting that representing the child in terms of their pre-adoption experiences may hamper the parent’s ability to see the child as an individual with a mind of their own. In the context of parenting children with disorders, there may be a risk of the child’s individual characteristics and personality being overshadowed by the disorder, or by the family’s experiences with diagnostic and support services. Based on the pattern observed in relation to placement-related descriptions in adoptive families, we expected that describing the child in terms of their disorder would correlate negatively with mind-minded descriptions.

There has been little research specifically on mind-mindedness in parents of children with developmental disorders: a recent review of mind-mindedness research did not identify any studies on this topic (see McMahon and Bernier 2017). Subsequently, Kirk and Sharma (2017) investigated mind-mindedness and parenting stress in parents of children with autism. Fifty-five mothers provided online descriptions of their children with ASD and 27 of those participants reported on an additional sibling without ASD. The findings showed that mothers were as likely to use mental state descriptors for children with ASD as for children without ASD, but they used more negatively-valenced mental attributes, such as “she is very anxious” or “he doesn’t understand other people’s feelings”. Negatively-valenced mental attributes correlated positively with the Difficult Child subscale of the Parenting Stress Index (Abidin 1995), suggesting that viewing one’s child as “difficult” is associated with negative attributions around their thoughts, feelings, and intentions. Similarly, Walker et al. (2012) assessed mind-mindedness in 24 parents of 3–5-year-olds referred to a Child and Adolescent Mental Health Service for emotional or behavioral disturbance, and found that parents in the clinical group provided a higher proportion of negative mental descriptions of their children (e.g., “spiteful”, “manipulative”). Unlike Kirk and Sharma’s (2017) findings, in this study overall mind-mindedness was found to be lower in the clinical group compared with a community sample of 25 parents and to correlate negatively with parenting stress within the clinical group. Understanding the benefits and challenges of taking a mind-minded or mentalizing stance towards children with developmental disorders is important to guide the kinds of emotional and practical support that may be required by families (Slade 2009).

In summary, the present study extended the investigation of mind-mindedness and its relation to parenting stress in parents of children with disorders. In line with previous research we predicted that (a) parents of children with developmental disorders would be more likely than those of TD children to describe them using negatively-valenced child descriptions, both with respect to mental and non-mental characteristics, (b) parenting stress would be higher in parents of children with developmental disorders than in parents of TD children, (c) mind-mindedness and positively-valenced descriptions would predict lower parenting stress, and negatively-valenced descriptions would predict higher parenting stress, and (d) disorder-related descriptions would be negatively related to mind-mindedness. Given equivocal findings in previous studies, we did not make a directional prediction about how overall mind-mindedness, or child descriptions with positive valence (e.g., kind, lovely, beautiful), would differ between groups. We included both genetic and developmental disorders to provide a wide range of parenting stress levels, since parents of children with ASD and ADHD have been found to experience greater stress levels than those of children with genetic disorders (Eisenhower et al. 2005).

## Method

### Participants

Participants were 235 parents (214 mothers, 21 fathers) of children aged between 2 and 18 years. Participants were recruited via schools, social media sites (Facebook, Twitter), charity and support group newsletters and websites, and researchers’ personal contacts. Parents were recruited if they had a typically-developing child (TD) (n = 90, 47 boys), or a child with a developmental disorder: ADHD (n = 51, 42 boys), ASD (n = 24, 21 boys), Down’s Syndrome (n = 38, 18 boys), or 22q11.2 Deletion Syndrome (22q11.2DS) (n = 32, 13 boys). Three respondents were adoptive parents of the child: 1 in the ADHD group, 1 in the Down’s Syndrome group, and 1 in the 22q11.2DS group. One respondent of a child in the TD group was a step-parent. For parent education level, the responses were coded as 0: up to undergraduate (38.6%), 1: undergraduate degree (35.6%) and 2: postgraduate degree (24.5%).

### Materials and Methods

Ethical approval was obtained via the relevant university ethics committee, and testing was conducted in line with the British Psychological Society guidelines. All data for this study were collected via an online survey, which participants accessed via a link to a Google Form. The first page contained an information sheet, at the end of which consent to participate was requested. Where consent was granted, the participants then accessed a page of questions on the demographics of their family and child (See Table 1), followed by the describe-your-child measure to assess mind-mindedness (Meins et al. 1998), and finally a measure of parenting stress (Abidin 1995). Participants were asked to respond in English, but three responses were given in Greek. These responses were translated into English by a member of the research team who was blind to participant condition before being back-translated into Greek by an external translator blind to the experimental design. The Greek passages were compared against the English translations for similarity before the English translations were utilised in the study.

#### Mind-Mindedness

Participants accessed a page which invited them to “Please describe your child in the space below”. No further instructions were given, so that the spontaneous focus of the parent could be assessed. Coding proceeded in line with Meins and Fernyhough’s (2015) mind-mindedness manual. Two coders who were familiar with the overall aims of the study but blind to the specific hypotheses, and who were a priori blind to which disorder each child had, divided the responses into individual attributes describing the child. The attributes were then coded as belonging to one of six categories: mental, behavioral, physical, general, self-referential, and disorder-related.

Mental attributes were related to the child’s mental life (e.g., “thoughtful”), interests (e.g., “he likes animals”), imagination or intellect (e.g., “bright”). Comments on knowledge, metacognition, or emotions (e.g., “can be very loving at times”) were also included. Behavioral attributes referred to the child’s behavior and interactive style. Descriptors like “funny”, “breaks things”, “boisterous” fell into this category. Physical attributes included comments on the child’s physical appearance (e.g., “beautiful”), age, or position in the family (e.g., “she is an only child”). Self-referential attributes were related to descriptions that primarily refer to the parent rather than the child (e.g., “he makes me smile”). General attributes included any comments that did not fall into the above-mentioned categories (e.g., “My daughter is a lovely little girl”). The final category of disorder-related comments is not included in the coding manual (Meins and Fernyhough 2015), but was developed for the current study to assess descriptions focused specifically on the child’s disorder (e.g., “Pretty mild on the spectrum”, “Ritalin has helped her with her learning”). Note, however, that if a description mentioning the disorder focused on the child’s mental or emotional experience of the disorder, it was coded as mental (e.g., “She doesn’t like people to know she has a learning difficulty”).

The mind-mindedness score was derived from the number of mental attributes expressed as a proportion of the total number of descriptions, and the disorder-related descriptions score was calculated in the same way. Inter-rater reliability was established by double-coding a random selection of around 20% of the participants’ responses; κ = .78 across the six categories.

Additionally, the emotional valence of all descriptions was coded. Demers et al. (2010) applied valence coding only to mental attributes, but because we were interested in valence across all attributes we coded each description of the child as positive, negative, or neutral. For example, a mental attribute such as “creative” was considered a positive mental description. A behavioral attribute such as “being rude” was coded as negative. A physical attribute such as “he is tall” was neutral in valence. Positive, negative, and neutral descriptions were calculated as a proportion of the total descriptions, with around 20% of cases being double-coded; κ = .67. Therefore, we could obtain the proportion of negative mental descriptions and positive mental descriptions, as well as negative and positive non-mental descriptions (i.e., non-mental descriptions comprised an aggregate of general, physical, behavioral, self-referential, and disorder-related descriptions). Positive and negative mental and non-mental scores were expressed as a proportion of all descriptions. Positive and negative proportion scores were not the inverse of each other, given that some descriptions were also coded as neutral. Neutral scores were not considered in the analyses.

#### Parenting Stress

Parents completed the Parenting Stress Index-Short Form (PSI-SF, Abidin 1995), a 36-item questionnaire in which parents are asked to indicate their agreement with a range of statements such as: “My child gets upset easily over the smallest thing” and “My child does a few things which bother me a great deal” on a 5-point Likert scale from strongly agree (1) to strongly disagree (5). The items were reverse scored so that higher raw scores indicated higher stress. The Total Stress Raw Score, with a possible range of 36–180, was used to provide an indication of the overall level of parenting stress: higher scores indicated greater stress levels. The internal reliability of the Total Stress score in this study was α = .96.

## Results

### Descriptive Statistics and Preliminary Analyses

One participant from the ASD group did not answer the describe-your-child question and therefore their information was removed from the dataset. Furthermore, there was one non-biological parent in the typically-developing (TD) group with an outlier score on the PSI-SF. This result created an association not reflected in the rest of the data, therefore this case was excluded from all analyses. On the PSI-SF, participants who failed to answer one question had the missing score prorated by calculating the mean for that subscale and using this number as the missing score and were still included in the analysis (Abidin 1995). Four participants who failed to answer two or more questions were excluded from analyses involving the PSI-SF (one parent from the ADHD group, one from the Down’s syndrome group, and two from the TD group). This left complete data from 233 participants. Three of these participants (1.3%) declined to report their education level, but their data for the other measures were retained in the analyses.

Initial analyses were run to investigate (a) the normality of the data, and (b) whether demographic variables were associated with the mind-mindedness or PSI-SF scores. The PSI Total score was not normally distributed for the TD (skew = .82, *SE* = .26; kurtosis = − .035, *SE* = .51) or Down’s Syndrome (skew = 1.35, *SE* = .39; kurtosis = 2.43, *SE* = .76) groups. The proportion of mental descriptions was not normally distributed for the ASD (skew = 1.01, *SE* = .48; kurtosis = .77, *SE* = .94) or TD (skew = − .02, *SE* = .26; kurtosis = −.47, *SE* = .51) groups, or in any of the groups for the positive and negative non-mental descriptions score. However, the *F*-test is robust to violations of the assumption of normality as long as there are at least 20 degrees of freedom for error (Tabachnick and Fidell 2007) so data were not transformed.

Demographic variables are presented in Table 1. PSI scores were positively correlated with the age of the child, *r*(227) = .22, *p* = .001, negatively correlated with the parent’s education level, *r*_*s*_(224) = − .20, *p* = .003, and were significantly higher for parents of boys than girls (*M*_boys_ = 83.01, *SD* = 29.06; *M*_girls_ = 73.35, *SD* = 26.22), *t*(227) = 2.57, *p* = .011. Mind-mindedness was not associated with parent age, parent education, or child gender. However, the proportion of negative mental descriptions was positively correlated with the age of the child, *r*(231) = .25, *p* < .001, negatively correlated with parents’ education level, *r*_*s*_(228) = − .18, *p* = .006, and was higher for boys than girls (*M*_boys_ = .06, *SD* = .09; *M*_girls_ = .03, *SD* = .07), *t*(231) = 2.75, *p* = .006. Positive mental descriptions were not associated with the demographic variables. The proportions of non-mental positive and negative descriptions were not associated with demographic variables and did not differ between groups. Therefore, all subsequent analyses controlled for child age, child gender, and parent education level.

There was a significant group difference in the number of children in families, *F*(4, 228) = 7.09, *p* < .001. Games-Howell posthoc tests showed that families with children with Down’s Syndrome tended to be larger (*M* = 1.79, *SD* = 1.14) than families of children with 22q11.2DS (*M* = 1.13, *SD* = .83) and TD children (*M* = .87, *SD* = .83). No other contrasts were significant. Therefore, subsequent analyses also controlled for the number of children in families.

### Differences in Mind-Mindedness and Valence Between Groups

**Fig. 1:**
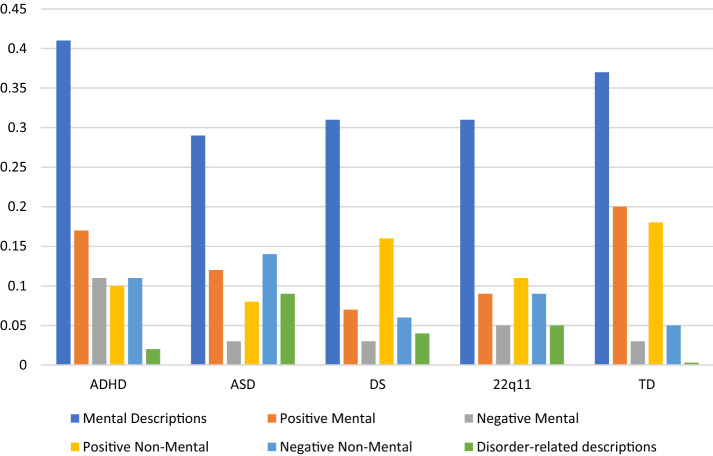
Mean proportion scores for child descriptions in each diagnostic group

We examined overall levels of mind-mindedness in our sample to establish whether there were differences between the diagnostic groups. Figure 1 displays the proportion scores for the child description variables used in the analyses. An ANCOVA was run across the five diagnostic groups, with proportion of mental descriptions as the dependent variable, diagnostic group as the fixed factor, and covariates of child age, child gender, parent education level and number of children in the family. There was no main effect of diagnostic group, *F*(4, 221) = 1.89, *p* = .114.

Next, we tested the valence of the mental descriptions used. We tested whether the proportion of negative mental descriptions was different between diagnostic groups. An ANCOVA was run identical to the previous model, but with the proportion of negative mental descriptions as the dependent variable. There was a main effect of group, *F*(4, 221) = 7.22, *p* < .001. Posthoc comparisons using Sidak correction showed that the ADHD group had higher proportions of negative mental descriptions than the Down’s Syndrome group (*p* = .002), ASD group (*p* = .001), and TD group (*p* < .001). No other comparisons were significant. The same model was run with positive mental descriptions as the dependent variable, which also showed a main effect of diagnostic group, *F*(4, 221) = 6.04, *p* < .001. Posthoc comparisons showed that the Down’s Syndrome and 22q11.2DS groups had significantly lower levels of positive mental descriptions than the TD group (*p* < .001 and *p* = .011, respectively). No other comparisons were significant.

Next, we tested for group comparisons in the use of negative, non-mental descriptions. An ANCOVA was again run across the five groups, identical to the previous model, with proportion of negative, non-mental descriptions as the dependent variable. There was a main effect of group, *F*(4, 221) = 3.20, *p* = .014. Posthoc tests using Sidak corrections showed that there was a marginally significant difference between the ADHD group and the TD group (*p* = .083), and between the ASD group and the TD group (*p* = .055), with both the ADHD and ASD group having higher proportions of negative, non-mental descriptions. We also tested the same model with the proportion of positive, non-mental descriptions as the dependent variable. There was a main effect of group, *F*(4, 221) = 4.12, *p* = .003, and the posthoc comparisons showed that there were significant differences between the TD group and the ADHD group (*p* = .028) and ASD group (*p* = 0.025), with the TD group having higher levels of positive, non-mental descriptions. No other comparisons were significant.

### Parenting Stress And Its Relation To Emotional Valence And Mind-Mindedness

Table 1 shows the means and standard deviations of the scores on the PSI and the describe-your-child measure. Parenting stress was highest for the ASD and ADHD groups and lowest for the TD group, consistent with previous research. An analysis of covariance (ANCOVA) was run to test for differences in parenting stress between the diagnostic groups, with PSI Total stress score as the dependent variable, child diagnosis as the fixed factor and covariates of child gender, child age, parent education level, and number of children in the family. There was a main effect of diagnostic group on PSI score after controlling for these covariates, *F*(4, 217) = 34.14, *p* < .001. Posthoc comparisons using Sidak correction showed that the parents of children with ASD or ADHD reported significantly higher stress than children in all other groups (*ps* < .001); there was no significant difference in stress levels between the parents of children with ASD and the parents of children with ADHD. Parents of children with 22q11.2DS reported higher stress than parents of TD children (*p* = .010). No other contrasts were significant.

**Table 1:** Descriptive statistics for parenting stress, mind-mindedness, and demographic variables

	ADHD	ASD	DS	22q11.2DS	TD
	*M*(*SD*) range	*M*(*SD*) range	*M*(*SD*) range	*M*(*SD*) range	*M*(*SD*) range
PSI-SF total	104.74 (28.24) 49–167	105.30 (30.00) 56–148	69.24 (16.20) 45–118	78.13(19.39) 48–119	62.07 (17.07) 40–108
Positive descriptions	0.27 (0.20) 0–0.72	0.19 (0.22) 0–1	0.23 (0.20) 0–0.71	0.20 (0.20) 0–1	0.38 (0.22) 0–1
Negative descriptions	0.22 (0.18) 0–0.63	0.16 (0.19) 0–0.67	0.08 (0.13) 0–0.44	0.13 (0.20) 0–0.77	0.08 (0.14) 0–1
Mental descriptions	0.41 (0.22) 0–1	0.29 (0.27) 0–1	0.31 (0.21) 0–0.75	0.31 (0.20) 0–0.71	0.37 (0.21) 0–0.80
Positive mental	0.17 (0.15) 0–0.57	0.12 (0.22) 0–1	0.07 (0.09) 0–0.33	0.09 (0.09) 0–0.40	0.20 (0.17) 0–0.75
Negative mental	0.11 (0.13) 0–0.50	0.03 (0.06) 0–0.20	0.03 (0.06) 0–0.20	0.05 (0.06) 0–0.18	0.03 (0.06) 0–0.25
Positive non-mental	0.10 (0.13) 0–0.46	0.08 (0.11) 0–0.40	0.16 (0.17) 0–0.66	0.11 (0.15) 0–0.60	0.18 (0.15) 0–0.67
Negative non-mental	0.11 (0.14) 0–0.63	0.14 (0.17) 0–0.67	0.06 (0.10) 0–0.44	0.09 (0.18) 0–0.66	0.05 (0.13) 0–1
Disorder-related descriptions	0.02 (0.09)	0.09 (0.22)	0.04 (0.12)	0.05 (0.10)	0.003 (0.03)
Child age	8.5 (3.8)	7.7 (5.0)	6.7 (4.2)	7.2 (5.5)	5.9 (4.6)
Parent education	0.67 (0.75)	0.64 (0.79)	0.76 (0.71)	0.97 (0.78)	1.01 (0.82)
Child gender (M; F)	42; 9	20; 3	18; 20	13; 19	47; 42

Next, we tested whether mind-mindedness predicted levels of parenting stress. Zero-order correlations for this model and the next were run to examine relations between the variables (Table 2). Multicollinearity checks were conducted. First, a hierarchical linear regression was conducted with PSI-SF as the dependent variable (Table 3). The co-variates and child diagnosis were included in the first step of the model. Mental descriptions (mind-mindedness) were included in the second step of the model. The first step of the model predicted 28% of the variance in parenting stress: child diagnosis was a significant predictor, and child age was marginally significant. The second step predicted an additional 1% of the variance: this change was significant (*p* = .043) and child diagnosis and mind-mindedness were significant predictors. The final model predicted 29% of the variance in parenting stress.

**Table 2:** Zero-order correlations between parenting stress, mind-mindedness and valence

	2	3	4	5	6
1. PSI-SF Total	− .27***	.34***	− .09	− .26***	.32***
2. Positive non-mental descriptions		− .18**	− .18**	.03	− .16*
3. Negative non-mental descriptions			− .27***	− .17**	.06
4. Mental descriptions				.58***	.30***
5. Positive mental descriptions					− .09
6. Negative mental descriptions					

Description scores are proportions of all descriptions used

*PSI* Parenting Stress Index

*p < .05, **p < .01, ***p < .001

**Table 3:** Summary of hierarchical regression analysis for variables predicting PSI scores

	*b*	SE B		B	*p*
Model 1					
Step 1					
Constant	95.14	5.22			.000
Child age	.70	0.38		.11	.066
Child gender	− 4.38	3.37		− .08	.195
Parent education	− 2.34	2.18		− .07	.283
Number of children	.90	1.71		.03	.600
Child diagnosis	− 7.81	1.08		− .45	.000
	R^2^ = .28, *F*(5, 220) = 16.97, *p* < .001				
Step 2					
Constant	100.91	5.91			.000
Child age	.66	0.37		.11	.078
Child gender	− 4.91	3.36		− .09	.145
Parent education	− 2.39	2.16		− .07	.270
Number of children	.93	1.70		.03	.582
Child diagnosis	− 7.83	1.07		− .45	.000
Mind-mindedness	− 15.03	7.40		− .12	.043
	∆R^2^ = .01, *F*(6, 219) = 15.03, *p* < .001				
Model 2					
Step 1					
Constant	95.14		5.22		.000
Child age	.70		0.38	.11	.066
Child gender	− 4.38		3.37	− .08	.195
Parent education	− 2.34		2.18	− .07	.283
Number of children	.90		1.71	.03	.600
Child diagnosis	− 7.81		1.08	− .45	.000
	R^2^ = .28, *F*(5, 220) = 16.97, *p* < .001				
Step 2					
Constant	93.45		5.58		.000
Child age	.33		0.35	.05	.354
Child gender	− 5.14		3.12	− .09	.101
Parent education	− 1.46		2.01	− .04	.468
Number of children	1.35		1.56	.05	.387
Child diagnosis	− 5.75		1.05	− .33	.000
Positive mental descriptions	− 25.75		9.53	− .15	.007
Negative mental descriptions	42.29		18.88	.13	.026
Positive non-mental descriptions	− 26.02		10.38	− .14	.013
Negative non-mental descriptions	46.84		10.66	.24	.000
	∆R^2^ = .13, *F*(9, 216) = 16.64, *p* < .001				

Next, we tested whether emotional valence predicted levels of parenting stress. A hierarchical linear regression was conducted with PSI-SF total as the dependent variable (See Table 3). Multicollinearity checks were conducted. Again, the covariates and child diagnosis were included in the first step of the model. Negative and positive mental descriptions, and negative and positive non-mental descriptions were included in the second step of the model.

As above, the first step of the model predicted 28% of the variance in parenting stress. The addition of positive and negative mental descriptions and positive and negative non-mental descriptions in the second step of the model explained an additional 13% of the variance. The final model explained 41% of the variance in PSI-SF score, with child diagnosis, negative mental descriptions, positive mental descriptions, negative non-mental descriptions, and positive non-mental descriptions as significant predictors. Positive descriptions predicted lower PSI scores while negative descriptions predicted higher PSI scores. Both negative and positive mental descriptions contributed independently towards parenting stress levels.

### Does Focus on Symptomatology Relate to Mind-Mindedness?

Only the four developmental disorders groups were included in this analysis. Due to the non-normal distribution, parametric and non-parametric tests were run but they showed similar results, therefore parametric results are reported for ease of interpretation of effect sizes. Disorder-related descriptions were used relatively infrequently as a proportion of all descriptions across the clinical groups (*M* = .05, *SD* = 0.13, Range = 0–1). The proportion of disorder-related descriptions was negatively correlated with the proportion of mental descriptions, *r*(144) = − .33, *p* < .001, and with the proportion of positive mental descriptions, *r*(144) = − .21, *p* = .011, but was unrelated to negative mental descriptions, *r*(144) = − .12, *p* = .168. This demonstrates that parents’ tendency to describe the child with reference to their disorder was associated with a lesser focus on the child’s overall mental and positive mental characteristics.

## Discussion

The results of this study showed that parents of children with developmental disorders did not have lower levels of mind-mindedness than parents of TD children, but in some groups used more negative descriptions of their children, both with regard to mental (ADHD group) and non-mental (ASD group) descriptions. Certain groups also experienced higher levels of parenting stress: parents of children with ASD and ADHD had higher stress than all other groups, while parents of children with 22q11.2DS had higher stress levels than parents of TD children, and parenting stress levels were predicted by the valence (positive and negative) of their mental and non-mental child descriptions. The present study also found that parents who described their children with reference to their disorder showed lower levels of mind-mindedness. While we had not made a specific prediction about positive child descriptions, we found that parents of TD children used the highest levels of positive descriptions for both non-mental and mental descriptions, though the latter were not significantly higher than the ADHD and ASD groups.

The first finding was in line with our predictions, showing that negative valence of child descriptions was more common in children with developmental disorders than children without, both for the mental and non-mental descriptions. In particular, children with ADHD were described most negatively for mental descriptions. Previous research has highlighted the specific challenges of parenting children with ADHD. In a study of mothers and children with ADHD, Psychogiou et al. (2008) reported that children’s ADHD symptoms were positively correlated with mothers’ negative comments about them on a free response task similar to the describe-your-child measure used in the present study. Interestingly, our results showed no differences in mind-mindedness as a function of children’s diagnostic group, and levels of mind-mindedness were in fact highest in the ADHD group. However, these parents also scored highest for negative mind-minded descriptions, which suggests that parents of children with ADHD may tend to view their children’s behavior as intentional or wilful, or to focus on their cognitive deficits (Ringer et al. 2019). Parents of children with ASD and ADHD also used the highest levels of negative non-mental descriptions. These findings emphasize the importance of considering the valence of child descriptions, particularly in populations of children with developmental disorders. Negative perceptions of children are regularly found to relate to higher distress for parents, poorer parent–child interaction and increased child behavior problems (Dadds et al. 2003; Deater-Deckard et al. 2005; Harrison and Sofronoff 2002; Hoza et al. 2000), and to moderate the relation between child behavior and parental reactions (Johnston and Patenaude 1994; Johnston and Ohan 2005; Waltzer et al. 2019).

Nevertheless, parents of children with ADHD and ASD did not differ from those of TD children with respect to their use of positive mind-minded descriptions. Positive mental descriptions were lower in the groups with genetic disorders (Down’s Syndrome and 22q11.2DS syndrome) than in the other groups. This may stem from the fact that learning disabilities are intrinsic to these disorders, whereas they are not to ASD and ADHD (APA 2013; Goodwin et al. 2017; Neece et al. 2012). It may be that parents of children with genetic disorders are less likely to focus on positive aspects of cognitive or intellectual functioning when thinking about their children. In the case of 22q11.2 syndrome, difficulties with intellectual functioning and high anxiety symptoms can often feature (Angkustsiri et al. 2012), and cognitive developmental outcomes are usually a key concern for parents of children with this disorder (Swillen and McDonald-McGinn 2015). Previous qualitative research has identified that children’s developmental delay relative to peers can be a source of anxiety and sadness for parents of children with Down’s Syndrome (Pillay et al. 2012). Parents of children with these genetic disorders may therefore be less inclined to focus on cognitive strengths or other positive mental qualities in their children.

However, negative mental descriptions were not higher in the 22q11.2DS and Down’s syndrome groups compared to the others, showing that parents did not appear to focus on problematic aspects of cognitive functioning or other mentalistic attributes. Indeed, Pillay et al. (2012) also found that parents’ dismay at developmental delay was often matched by pride in their children’s accomplishments. Furthermore, we found that overall mind-mindedness was not any lower than in the other comparison groups, suggesting that parents did not avoid describing their children with reference to mental states. More detailed research on parental attributions towards children with genetic disorders would be helpful to elucidate the nuances behind these findings.

Also in line with predictions, we found that parenting stress levels were significantly higher in children with developmental disorders: specifically parents of children with ASD, ADHD, and 22q11.2DS. Parents of children with Down’s Syndrome did not report higher stress levels than TD children. This is partly in line with previous research suggesting that parenting children with neurodevelopmental disorders is more stressful than parenting children with genetic disorders (Eisenhower et al. 2005; Pottie and Ingram 2008). Additional factors associated with 22q11.2 deletion syndrome may explain the higher levels of stress in these parents than in parents of children with Down’s Syndrome—as a rarer, less recognized syndrome, parents are often faced with invalidating responses in social and health settings which can contribute to anger and stress (Goodwin et al. 2017; Hallberg et al. 2010).

Our prediction that parenting stress would be predicted by the valence of parents’ mental and non-mental descriptions of their children was also supported. Negative non-mental child descriptions predicted higher parenting stress, and positive non-mental descriptions predicted lower parenting stress. Mind-mindedness was also a significant predictor of parenting stress, in line with McMahon and Meins (2012), suggesting that being able to tune in to the child’s mind is associated with lower stress levels. In addition, negative mind-mindedness (i.e., using descriptions such as ‘quick to anger’) was predictive of higher parenting stress. This is in line with Walker et al.’s (2012) findings that parents of 3–5-year-olds attending a clinic for emotional and behavioral problems had higher levels of negative mind-mindedness than a community comparison group, which correlated positively with parenting stress. It also coheres with Kirk and Sharma’s (2017) finding that parenting stress in ASD was positively correlated with negative mind-mindedness.

The current findings suggest that having a tendency to perceive and understand one’s child’s thoughts, feelings, and motivations in negative terms is associated with feeling more stressed as a parent, irrespective of diagnosis. The connection between parenting stress and mind-mindedness is likely to be complex and bidirectional. Stress levels will influence how readily parents can consider and reflect on their child’s perspective, but negative parental attributions or mind-mindedness may lead to more coercive parenting, withdrawal from the child, and thus a more stressful experience (Bugental and Johnston 2000; Danforth and Diller 2020; Harrison and Sofronoff 2002; Holahan et al. 2005; Kirk and Sharma 2017).

Our findings have some clinical implications, as they suggest that supporting parents to read their children’s mental lives in less negative ways may help with feelings of stress. Interventions that seek to alter or correct negative attributions may thus offer a means to reduce parenting stress (Bussanich et al. 2017; Hassall and Rose 2005; Johnston and Ohan 2005). Research has addressed the topic of parental attributions towards challenging behavior in the field of child abuse and emotional/behavioral disorders (e.g., Dadds et al. 2003; Sawrikar et al. 2018), but research in this area could also be beneficial for the field of developmental disorders. For instance, clinical interventions that attempt to render a child’s behavior explicable and developmentally-based have been shown to reduce parenting stress in ADHD (e.g. Foubister et al. 2020) and ASD (Keen et al. 2010).

We also found that positive mind-mindedness (descriptions such as ‘loving’ or ‘clever’) was associated with lower parenting stress across the diagnostic groups, suggesting that there may be a protective effect of being able to attribute positive mental qualities to the child. This finding is in line with Demers et al. (2010) and McMahon and Meins (2012) who also found that having a positive representation of the child’s mental life was negatively associated with parenting stress. This supports mainstream parenting approaches (e.g., Kabat-Zinn and Kabat-Zinn 2014; Lansbury 2014) and parenting programs (e.g., Parents Plus, see Carr et al. 2017) that aim to build parents’ capacity for a strong, positive understanding of the child and reasons for difficult behavior, rather than endorsing formulaic behavior management strategies. It may be the case that an enduring positive view of the child can maintain warmth in the relationship and protect against the challenges caused by disruptive behavior or other needs (Deater-Deckard et al. 2005; Hughes et al. 2017), which can be foregrounded in children with developmental disorders. For example, Solomon et al. (2008) found that, following parent–child interaction therapy for children with ASD, parent perceptions of child behavior problems and child atypicality reduced, a change which resulted in increased positive parental affect during interaction with the child. Interventions that attempt to improve positive mind-mindedness may therefore be beneficial in the field of developmental disorders.

Finally, in line with our prediction, we found that parents whose descriptions focused on aspects of their child’s diagnosis (e.g., ‘has had open heart surgery’, ‘sees a speech therapist’) were less likely to describe their children with reference to their mental states. The prominence of these descriptions makes sense, given parents’ histories of describing their children’s difficulties during assessments, caring for their particular needs, and often advocating for their children in health, social, and educational settings (e.g., Farkas et al. 2019). Nevertheless, could this predominance potentially affect the relationship between the parent and child? Clinically, the concept of ‘diagnostic overshadowing’ describes the tendency for professionals to attribute aspects of people’s behavior or mood to their primary diagnosis, thus failing to notice the presence of comorbid conditions (e.g., a depressed person with ASD may spend a lot of time in their bedroom, but this behavior is interpreted as part of their ASD rather than evidence of depression, also see Nash 2013).

It is possible that a similar ‘overshadowing’ process could occur between parents and children with a developmental disorder, whereby parents may become focused on the diagnosis or problematic symptoms thereof, rather than on their child’s personality and character. This is by no means universal—Kuhaneck et al. (2010) reported findings from a qualitative study of parents of children with ASD in which parents described the benefits of “lifting the restraints of labels” (p. 346) and not losing sight of their own children’s personalities. Nevertheless, the findings from the current study show that mind-mindedness was negatively correlated with disorder-related descriptions. As mind-mindedness is associated with a range of positive child outcomes, at least in TD samples (e.g., McMahon and Bernier 2017), an important clinical implication of this research is to ensure that parents are supported to think and talk about their child with a developmental disorder with reference to positive, mental state attributes. For example, Attwood (2006) advises clinicians conducting assessments with adults with suspected ASD to collate family views on, and communicate about, the person’s qualities as well as their diagnosis-related difficulties, in order to retain a focus on the person as a unique individual. Of course, the disorder and the child’s psychological qualities may be intrinsically connected, but the important point is for parents not to lose sight of their child’s individuality (McMahon and Meins 2012). Further research to explore how parents balance their view of the child as an individual against a focus on medical or educational needs, challenging behavior, or mental health is needed, as well as to develop and evaluate interventions that promote mentalizing towards children with developmental disorders.

The results of the present study should be interpreted in light of a number of limitations. The children’s diagnoses were not independently verified by the study team but were reported by parents, which introduces the possibility of errors in diagnoses. Future studies could include diagnostic measures or replicate these investigations in clinical settings where diagnoses are verified by clinicians. We did not measure the severity of children’s difficulties through symptom scales such as the Strengths and Difficulties Questionnaire (Goodman 1997), as parent-reported behavioral difficulties tend to correlate very highly with parenting stress (McSherry et al. 2019). Future research should examine whether the severity of children’s clinical symptoms is associated with parental mind-mindedness. Furthermore, the groups were not evenly matched on sample size, and we did not consider additional variables such as children’s IQ, adaptive functioning or language ability, which would also influence the experience of parenting.

However, a strength of the study is the inclusion of children with both chromosomal and developmental disorders. Parenting children with ASD and ADHD may be more challenging and stressful than parenting children with genetic disorders for a number of reasons. As ASD and ADHD are diagnosed through behavioral symptoms rather than genetic testing, parents lack objective proof of their children’s disorder and may have queried over the years before diagnosis whether their children’s difficulties were wilful or intentional. In addition, parents may find lack of understanding on the cause and prognosis of their child’s disorder difficult to tolerate (Dale et al. 2006) and may suffer from the high levels of public stigma that surround neurodevelopmental disorders, particularly ADHD (Mikami et al. 2015). In the current study we found that parenting stress for parents of children with Down’s Syndrome was no different to the TD group. We also found low levels of positive mental descriptions of children with DS and 22q11.2 syndrome, as discussed above.

The findings provide further evidence that the experiences of parenting children with genetic versus neurodevelopmental conditions may be distinct in particular ways. Future research is needed to examine these distinctions. In addition, further research is warranted to examine whether early mind-mindedness in parents of children with developmental disorders is similarly associated with positive child outcomes as has been found with TD children, perhaps using studies with infant siblings of children with neurodevelopmental disorders and young children diagnosed with genetic disorders.
